# Seroprevalence and risk factors of recent infection with hepatitis E virus during an acute outbreak in an urban setting in Chad, 2017

**DOI:** 10.1186/s12879-018-3194-6

**Published:** 2018-06-26

**Authors:** Larissa Vernier, Annick Lenglet, Boris M. Hogema, Ali M. Moussa, Cono Ariti, Simone Vollmer, Andrea Irwin, Prince Alfani, Sibylle Sang, Charity Kamau

**Affiliations:** 1 Médecins sans Frontières, Operational Center Amsterdam (OCA), Quartier Aeroport, A1, Rue 1039, Porte 405, BP30, N’Djamena, Chad; 2 Médecins sans Frontières, Operational Center Amsterdam (OCA), Plantage Middenlaan 14, 1018DD Amsterdam, the Netherlands; 3 Sanquin Diagnostic Services, Department of Virology, Plesmanlaan 125, 1066 CX Amsterdam, The Netherlands; 4 Ministry of Health, BP440 N’Djamena, Chad; 5 Medical Statistics, London School of Hygiene and Tropical Medicine, Keppel Street, London, WC1E7HT UK

**Keywords:** Hepatitis E virus, Chad, Seroepidemiologic studies, Risk factors, Disease outbreaks

## Abstract

**Background:**

From September 2016–April 2017, Am Timan, Chad, experienced a large HEV outbreak in an urban setting with a limited impact in terms of morbidity and mortality. To better understand HEV epidemiology in this context, we estimated the seroprevalence of anti-HEV antibodies (IgM and IgG) and assessed the risk factors for recent HEV infections (positive anti-HEV IgM) during this outbreak.

**Methods:**

A serological survey using simple random sampling was implemented in Am Timan at the tail-end of the outbreak (sample size aim = 384 household). Household members provided us with blood samples and household heads answered questions around water, sanitation and hygiene practices and animal ownership. Blood samples were tested for HEV IgG and IgM antibodies using Enzyme-Immune-Assay (EIA). We calculated weighted prevalence estimates and prevalence ratios (PRs) for possible risk factors for recent infection using multivariate Cox regression.

**Results:**

We included 241 households (1529 participants). IgM prevalence decreased with age: 12.6% (< 5 years) to 4.3% (> 15 years). IgG prevalence increased with age: 23.5% (< 5 years) to 75.9% (> 15 years). Risk factors for recent HEV infections included: sharing the sanitation facility with other HHs (PR 1.72; 95%CI: 1.08–2.73), not systematically using soap for HW (PR 1.85; 95%CI: 1.30–2.63) and having animals sleeping inside the compound (PR 1.69; 95%CI: 1.15–2.50).

**Conclusions:**

Evidence suggests that Am Timan was already highly endemic for HEV before the outbreak, potentially explaining the limited extent of the outbreak. Recent infection with HEV was linked to household level exposures. Future HEV outbreak response must include ensuring access to safe water, and reducing household level transmission through active hygiene and sanitation promotion activities.

**Electronic supplementary material:**

The online version of this article (10.1186/s12879-018-3194-6) contains supplementary material, which is available to authorized users.

## Background

Hepatitis E virus (HEV) is a common cause of acute viral hepatitis worldwide [1]. Genotypes 1 and 2 of HEV are spread through fecal-oral transmission (from contaminated water supplies) and have been implicated in outbreaks in Asia, Africa, Latin America and the Middle East. In contrast, infection with HEV genotypes 3 and 4 are of zoonotic origin. Although the true burden of HEV globally remains unknown, it has been estimated that around 20 million of people are infected by genotype 1 and 2 annually, resulting in 3.4 million cases of symptomatic illness, 70,000 deaths and 3000 stillbirths annually [2]. The disease is usually self-limiting but severe cases can develop into fulminant hepatitis which can lead to neurological sequelae, spontaneous abortions and sometimes death. The overall case fatality rate (CFR) is estimated between 4 and 30%, but has been documented to reach up to 40% in pregnant women, particularly during the third trimester [3, 4].

Large outbreaks of HEV have been previously reported from sub-Saharan Africa, mostly in refugee and internally displaced persons (IDPs) settings in Sudan, Uganda, Kenya, South Sudan and Ethiopia [5–11]. It is known that HEV genotype 1 was implicated in the outbreaks reported from Sudan, South Sudan and Uganda [12–14]. Risk factors for HEV infection that were identified in previous outbreaks included the consumption of chlorinated surface water, handwashing in communal handwashing facilities, storing drinking water in wide-mouthed containers and having other household members previously presenting with jaundice, suggesting a level of household transmission [6, 8, 15]. There is also evidence from the outbreak in Uganda between 2007 and 2009 that person-to-person transmission was implicated in the propagation of the outbreak [16].

Chad experienced an HEV outbreak in 2004 in Goz Amer refugee camp in which 672 cases and 21 deaths of acute jaundice syndrome (AJS) were reported between June and August [17]. Between September 2016 and April 2017, a large urban outbreak of HEV genotype 1e spread in Am Timan, Chad [18]. During the course of the outbreak, 1443 cases of AJS were reported through community-based surveillance activities, and 100/250 AJS cases were confirmed as HEV infected by the Assure® Hepatitis E IgM rapid diagnostic test (RDT) from MP Diagnostics [18]. The overall AJS attack rate (AR) was estimated at 2% and three pregnant women died (2% of all pregnant women with AJS and 20% of hospitalized ones). It should be noted that active case finding for AJS was in place in this outbreak which is a very sensitive case definition, thus likely overestimating the true number of actual HEV infections. In this outbreak, risk factors for confirmed HEV infection included having at least two children below the age of 5 years living in the household, having another household member with jaundice and not always washing hands before meals, but the latter two were of limited statistical significance [18].

The Am Timan outbreak, though it was the largest documented in an urban setting in Sub-Saharan Africa, had a limited impact in terms of morbidity and mortality [18]. Previous outbreaks have reported higher ARs and more dramatic epidemic curves (particularly the refugee/IDP camp outbreaks in South Sudan and Uganda) [13, 14, 19]. In previous HEV outbreaks in sub-Saharan Africa, the CFR ranged from 1.5 to 17.8% and from 12.5 to 42.1% in overall population and pregnant women, respectively [20]. The reasons for the different epidemiological profiles of outbreaks of this genotype of HEV in sub-Saharan Africa remain unclear. Therefore, the outbreak in Am Timan, Chad, presented an opportunity to determine the seroprevalence of anti-HEV antibodies (IgM and IgG) in different age groups in March–April 2017 (the tail-end of the outbreak) and to assess the risk factors for recent HEV infections. We expected that these results would add the available evidence around HEV genotype 1 transmission in acute outbreak settings.

## Methods

### Study setting

The study was implemented between March 22 and April 27 2017 in Am Timan in the eastern part of the Salamat region in Chad. It is a town of approximately 65,000 residents (estimates by Médecins Sans Frontières (MSF), December 2016) and is home to a large semi-nomadic population.

### Study design and sample size

We designed a cross-sectional population-based serological survey with a sample size large enough to precisely estimate seroprevalence estimates in three age groups: 0–4 years, 5–14 years and ≥ 15 years. In order to estimate a 50% level of seropositivity (IgM or IgG) with a simple random sampling (design effect = 1) and a precision of 5%, we required 382 persons per age group strata in order to have a representative sample. We estimated that the proportion of the population that represented these age groups was the following: 0–4 = 30%; 5–14 = 32%; ≥15 = 39% and the average household size was 5.5 persons. Thus, the number of households to include to reach this sample size in the least represented age group (0–4 years) was 232 households. Accounting for a refusal rate of 40%, we aimed to visit 384 households.

### Participants

For the purpose of a hygiene kit distribution in December 2016, we created a comprehensive list of all, approximately 11,000, households in Am Timan. All households were assigned a unique number that consisted of a block number and house number respective to their location in the town. We used simple random sampling without replacement to select 384 households from this list using Excel 10.

The head of the household was any member of the sampled household who was 18 years or older, who could give accurate information on the household members and household exposures, who identified as the head of the household and who was present at the time of the study team visit. Household members were considered eligible for inclusion in the survey if they: were resident of that household since September 2016 (start of the outbreak), had not been absent for more than 2 weeks since September 2016, had not received blood or blood products in the 3 months before the survey, were in a health condition that would allow us to draw blood and agreed to participate.

### Data collection

In each selected household, two questionnaires were administered: a household questionnaire and an individual questionnaire (Additional file 1: Household and Individual Questionnaires in French and Additional file 2: Household and Individual Questionnaires in FrenchEnglish). The household questionnaire was administered to the household head and contained questions related to practices around drinking water sources, water treatment, water storage, sanitation facility, handwashing practices and animal ownership at the time of the interview. Observations of the water storage containers, sanitation facilities, handwashing practices and ownership of soap were also carried out. Additionally, the household head was asked to report any cases and deaths from AJS that had occurred in his/her family since the start of the outbreak in September 2016. The individual questionnaire included questions on sex, age and history of self-reported symptoms (including jaundice) since September 2016. It was administered to each household member older than 7 years, and to the parent/caretaker of the children younger or equal to 7 years old. Both questionnaires were completed on tablets using Open Data Kit (ODK).

Each participating individual was also asked to provide four milliliters (mL) of venous blood. Blood was drawn in sterile vacutainer tubes with ethylene diamine tetra acetic acid (EDTA) anticoagulant by professional healthcare staff. In infants difficult to handle, we collected less volume of blood or collected capillary blood from the heel in rare instances. Samples were then transported to the hospital laboratory where they were centrifuged. Serum samples were transported to Sanquin Diagnostic Service (The Netherlands) for the laboratory investigations. Transportation and storage of blood samples was done using a cold chain (2-8 °C).

### Laboratory methods

All serum samples were tested for HEV IgG and IgM antibodies using Wantai Enzyme Immuno Assay (EIA) tests (Sanbio, Uden, the Netherlands) according to the instructions of the manufacturer. The IgM test is known to have a 99% specificity and a 75% sensitivity for clinical use which is the highest of the current commercial HEV serological tests available [21]. According to the manufacturer’s instruction, the sensitivity for the IgG test is 100%. The specificity cannot be calculated easily as there is no gold standard test available and individuals may inverse seroconvert due to waning antibody titers. By testing samples from 2 to 3 year old children and adults the sensitivity was estimated to be 97.8% [22]. We defined a recent HEV infection as any person with detectable anti-HEV IgM. We defined a past HEV infection as any person with detectable anti-HEV IgG who did not have any detectable anti-HEV IgM.

HEV IgM positive samples were first re-tested for HEV IgG and IgM antibodies after high-speed centrifugation (10 min, 10,000×g). Samples that were repeatedly tested HEV IgM reactive were subjected to PCR using an in-house test with a 95% limit of detection of 10.3 IU/ml, as described previously [23]. Genotyping was performed for all samples with a viral load > 2000 IU/ml. ORF1 and ORF2 regions were amplified and sequenced as described previously [23].

### Data analysis

Data analysis was performed using STATA version 12 and 13 (StataCorp, College Station, TX, USA). Overall and age-stratified prevalence estimates along with their 95% confidence interval (95%CI) were calculated for self-reported jaundice and HEV IgM and IgG antibodies. To infer the prevalence of the main health outcomes to the whole Am Timan population, adjusted prevalence estimates were calculated to account for clustering of cases within households. To be consistent with previous population distributions, the prevalence estimates were also weighted to account for the underrepresentation of children under 5 years (harder to get blood from) and men (more likely to be at work/absent during the interview and more likely to have travelled in the last 6 months and therefore not eligible for inclusion). Comparisons of prevalence estimates between age groups were performed by Chi-squared and Fisher’s exact tests.

To determine household-level and individual exposures associated with recent HEV infections, we conducted a risk factor analysis comparing people with recent HEV infections (IgM+) to people without evidence of any HEV infection (IgM-/IgG-). Individuals with past HEV infections (IgM-/IgG+) were excluded because we could not distinguish the past infections having occurred during this outbreak from those having occurred in the more distant past. Although the presence of animals sleeping inside the compound was assessed separately for several animals i.e. chicken, dogs, cats, cows, goats, donkeys, pigeons and horses, a single variable was created to determine the association between the presence of any animals sleeping inside the compound and recent HEV infections. Using univariate Cox regression models, we estimated unadjusted prevalence ratios (PR) with their respective 95%CI for each exposure presenting enough variation among the Am Timan population (no category was included that was below 5% of total sample size). To identify risk factors and calculate adjusted PRs, we included exposures fulfilling the following three criteria in a multivariate Cox regression model: a) *p*-value < 0.2 in the univariate analysis, b) sufficient outcomes in each category of the exposure variable and c) sensible in terms of disease control recommendations. Variables to be kept in the final model were selected using the backward stepwise selection method (significance level < 0.05). All our models were adjusted for age and sex. All analyses accounted for the clustered and weighted survey design using Taylor-linearised adjustments to the standard errors as described in Heeringa et al. (2017) [24] and implemented using the svy commands in Stata.

### Ethical considerations

The study was conducted in accordance with the Council for International Organisations of Medical Sciences (CIOMS) International Ethical Guidelines for Biomedical Research Involving Human Subjects and International Ethical Guidelines for Epidemiological Studies. The study protocol was approved by the Ethical Review Board of MSF (Study ID: 1646) and the Chadian Ministry of Health. Confidentiality and anonymity for participants were strictly respected for the duration of the study and in arising reports.

The head of the household was asked to provide informed written consent to conduct the household questionnaire and include household members in the study.

All available laboratory results were communicated back to participating individuals within 2 months after the end of data collection. All persons identified with acute infections were offered counselling and medical follow-up at the hospital if necessary.

## Results

### Overall findings

**Fig. 1:**
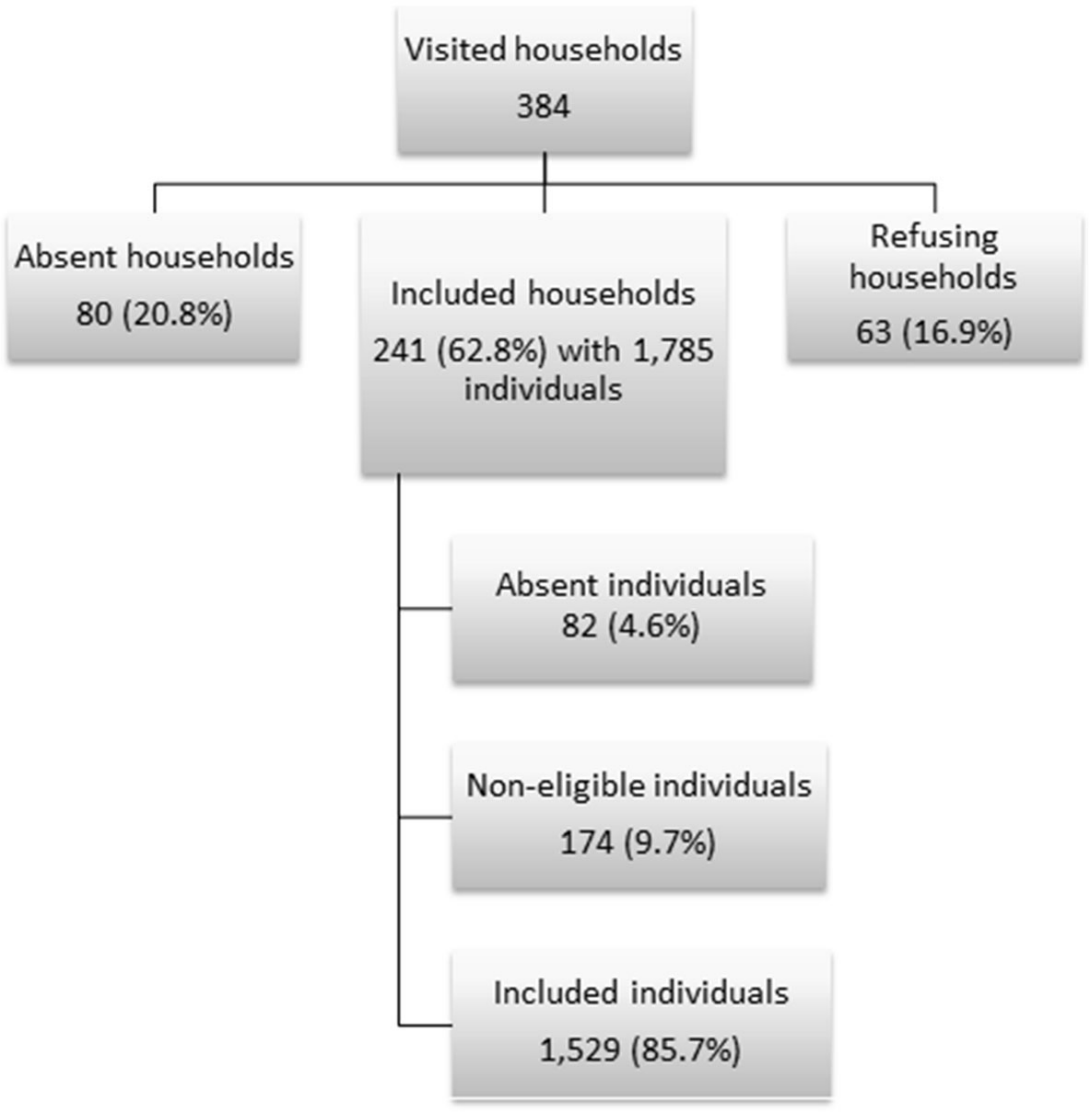
Flowchart of household and study participants

A total of 384 households were visited; 80 (20.8%) households were absent and 63 households (16.9%) refused to participate (Fig. 1). We included 241 HHs in the analysis (response rate: 63%). Households consisted of a median of 6 individuals (range: 1–37, mean = 7.4). Among the 1785 individuals living in the participating households, 174 (9.7%) were not eligible for inclusion in the study and 82 (4.6%) were absent on the day of the survey. We included 1529 participants in the final analysis.

The majority of the participating households (*n* = 143, 59.3%) relied on boreholes with or without an open tank as their source of drinking water. Most surveyed households (*n* = 228, 94.6%) reported that their water was chlorinated as this was being organized at all public water points as part of the HEV outbreak response. Most households (*n* = 184, 76.4%) used a pit latrine without a slab as their sanitation facility and of the 226 latrines (99.1%) that were inspected, 69.0% (*n* = 156) were visibly dirty with feces. Thirty-seven AJS cases were reported between September 2016 and the day of the interview in 30 (12.5%) HHs. The cumulative AR for self-reported AJS during the outbreak was 2.1 per 100 persons (37/1785). Of these, two died, resulting in a AJS mortality rate and CFR of 0.1% (2/1785) and 5.4% (2/37), respectively.

### Hepatitis E prevalence in Am Timan

**Fig. 2:**
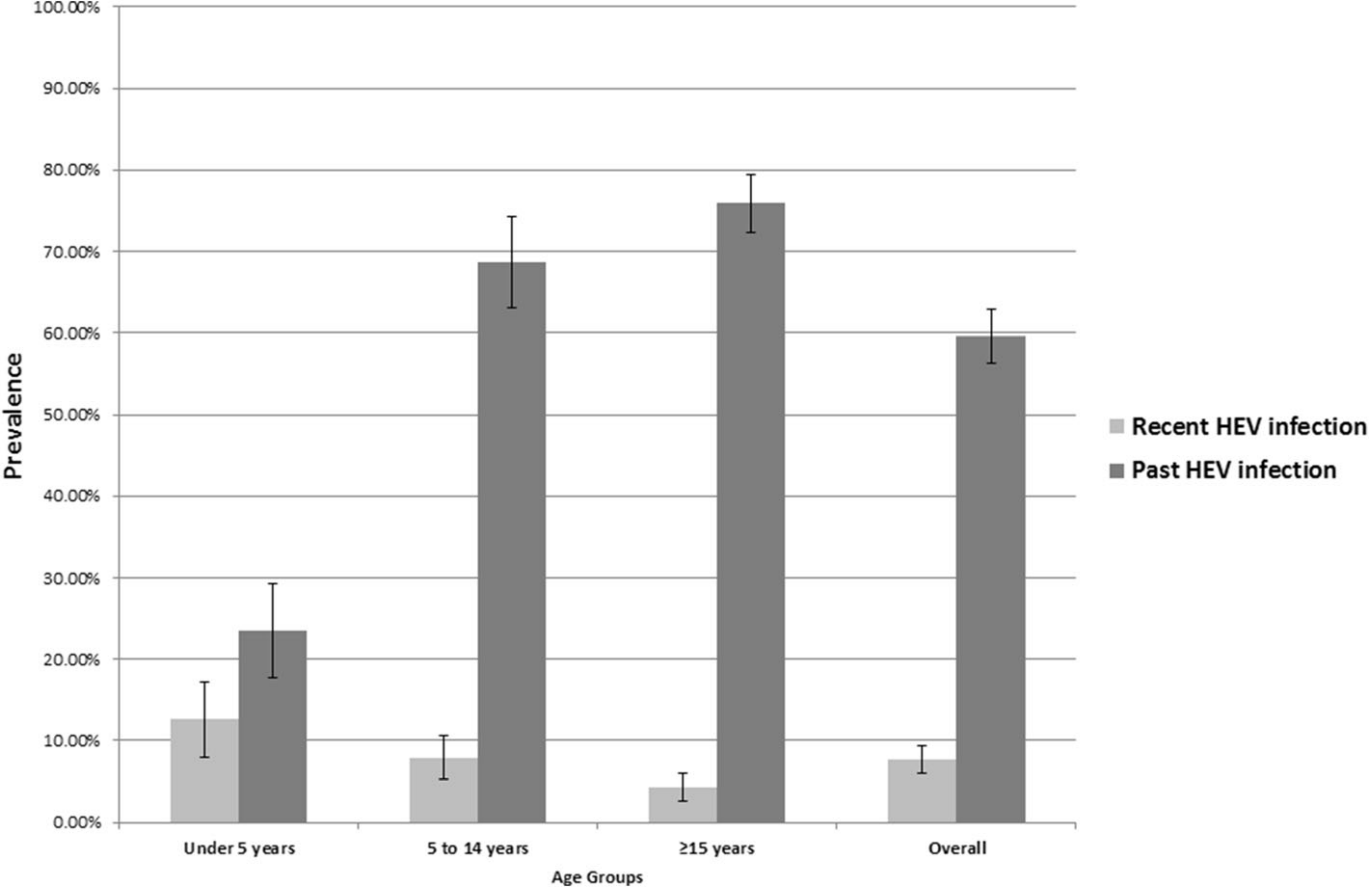
Adjusted seroprevalence of recent and past HEV infection by age group (*N* = 1494) Note: Recent HEV infection = anti-HEV IgM+ / IgG ± and past HEV infection = anti-HEV IgM- / IgG+.

The prevalence of recent HEV infection and past HEV infection was 7.7% (95%CI: 6.2–9.6) and 59.6% (95%CI: 56.3–62.8), respectively (Fig. 2; Additional file 3: Table S1). The prevalence of HEV recent infection was as high as 12.6% (95%CI: 8.7–17.9) among children under the age of 5 years and significantly decreased with age (Chi = 8.53; *p* < 0.01) (Fig. 2). On the contrary, the prevalence of past HEV infection significantly increased with age (Chi = 107.30; p < 0.01) and reached 75.9% (95%CI: 72.1–79.2) among individuals aged 15 years and older (Fig. 2). Recent infections were clustered in households with other recent infections; more than half of the recent infections (*n* = 58; 55.8%) were found in households with at least one more recent HEV infection.

The proportion of Am Timan population still susceptible to HEV was 32.7% (95%CI: 29.6–35.9). The proportion of susceptible persons significantly decreased with age (Chi = 82.18; p < 0.01). Among the population aged 5 years old and above, around 20% of the people were susceptible, whereas 63.9% (95%CI: 57.4–70.0) of the children under the age of 5 years old were still susceptible to HEV.

Out of 453 women aged ≥15 years, 451 (99.6%) reported about their pregnancy status; 44 (9.7%) women aged ≥15 years reported themselves as pregnant at the time of the survey. The prevalence of recent HEV infection among self-reported pregnant women was 2.3% (95%CI: 0.3–15.2).

### HEV genotype

Among the 104 recent infections, 11 (10.6%) were positive for HEV RNA using PCR; 90 (86.5%) were negative and 3 (2.9%) were indeterminate. Three HEV RNA positive samples underwent genotyping which identified genotype 1e virus which was homologous to HEV isolates from Sudan and Chad.

### Self-reported jaundice during outbreak and estimating HEV seroprevalence prior to the outbreak

Out of the 1527 persons included in the analysis, 30 self-reported having experienced AJS since September 2016. Of these, seven were persons with a recent infection (symptomatic rate: 7/104, 6.7%) and 19 persons had a past infection (symptomatic rate: 19/959, 2.0%). Both the IgM (Chi = 13.44, *p* < 0.01) and IgG (Chi = 4.95, *p* = 0.026) status were associated with self-reported AJS since September 2016.

The impact of the 2016–17 outbreak could not be directly inferred from our seroprevalence estimates, as past HEV infections could have occurred either early in this outbreak or before the beginning of it. However, under the assumption that 6.7% of recent infections with self-reported jaundice represent the true symptomatic rate of infection during this outbreak, we could extrapolate from the total number of jaundice cases that 388 HEV cases (104 recent infections and 284 past infections) had occurred since September 2016 (cumulative AR of 26.0% (388/1492)). In turn, this would mean that 675 past HEV infections occurred before September 2016. With this estimation, 45.2% of the population was immune before the start of this outbreak.

### Risk factors associated with recent HEV infections

We compared 104 individuals with recent HEV infections and 429 persons without any HEV infection (past or recent). The unadjusted analysis indicated that having at least one child < 5 years of age in the household, not systematically using soap during handwashing and having an animal sleeping inside the compound were associated with having a recent infection of HEV (Table 1). Sex was not associated with a recent HEV infection in the unadjusted and adjusted analysis. Compared to persons older than 14 years of age, there was weak evidence that being aged 5–14 years of age was associated with recent infection of HEV. No variables around household composition were retained in the adjusted analysis. Factors that remained associated with recent infection in the adjusted analysis included: sharing the sanitation facility with other households (PR 1.72; 95%CI: 1.08–2.73), not systematically using soap for handwashing (PR 1.85; 95%CI: 1.30–2.63) and having animals sleeping inside the compound (PR 1.69; 95%CI: 1.15–2.50).

**Table 1:** Risk factors associated with recent HEV infections in unadjusted and adjusted Cox regression analysis

Factor	Recent infections (*N* = 104)	No infection (*N* = 429)	Unadjusted PRs		Adjusted PRs	
			PR (95%CI)	*p*-value	PR (95%CI)	*p*-value
Age group [years]				0.08		0.04
0–4	33 (31.7%)	165 (38.5%)	0.84 (0.51–1.39)		0.88 (0.53–1.46)	
5–14	42 (40.4%)	125 (29.1%)	1.46 (0.89–2.39)		1.54 (0.94–2.51)	
≥15	29 (27.9%)	139 (32.4%)	Reference		Reference	
Sex						
Female	55 (52.9%)	222 (51.8%)	1.02 (0.72–1.45)	0.89	1.07 (0.77–1.48)	0.68
Household composition						
At least one child < 5 years old	92 (88.5%)	357 (83.2%)	1.88 (1.06–3.3)	0.03	^c^	
> 6 persons	84 (80.8%)	328 (76.5%)	1.42 (0.89–2.26)	0.14	^c^	
≥ 50% of the household with past HEV infection	53 (51.0%)	179 (41.7%)	1.37 (0.90–2.07)	0.14	^c^	
Water, sanitation and hygiene						
Wide-mouthed containers owned^a^	92 (88.5%)	400 (93.5%)	0.62 (0.33–1.16)	0.13	^c^	
Shared sanitation facility^b^	82 (80.4%)	287 (70.2%)	1.67 (0.88–3.16)	0.12	1.72 (1.08–2.73)	0.02
No handwashing point next to the sanitation facility^b^	67 (65.7%)	303 (74.1%)	0.72 (0.49–1.07)	0.11	^c^	
No systematic use of soap for handwashing	42 (40.4%)	107 (24.9%)	1.68 (1.14–2.48)	0.01	1.85 (1.30–2.63)	< 0.01
Animal sleeping inside the compound	35 (33.7%)	87 (20.3%)	1.65 (1.11–2.47)	0.02	1.69 (1.15–2.50)	0.01

^a^IgM + =104 / IgM- = 428; ^b^ IgM + =102 / IgM- = 409; ^c^Not kept in the final multivariate model

## Discussion

We have determined that the prevalence of anti-HEV IgM in April 2017 in Am Timan was relatively low across all age groups considering the outbreak (including in the most susceptible group i.e. persons younger than 5 years). This can be explained by the high levels of anti-HEV IgG determined across all age groups which would reduce the number of infections and by the fact that the study was carried out during the tail-end of the outbreak as suggested by surveillance data [18].

The prevalence of anti-HEV IgG was high and increased rapidly after 5 years of age. It is possible that prior to this outbreak, the population of Am Timan was already highly exposed to HEV. The low number of susceptible persons present in the population would therefore have been low when the outbreak started. It is also possible that the outbreak infected up to 67.3% of the population over the 7–8 months of the documented outbreak but that the IgM levels had already waned for the majority of these when the study was implemented. However, this would not explain the different IgM seroprevalence between children and adults. Comparatively, 9 months after an HEV outbreak onset in an IDP camp in Kitgum, Uganda (2007–2009), 21.1% of the population tested positive for anti-HEV IgG [7]. Similar low levels of IgG levels were identified in a survey conducted 4 months after an HEV outbreak onset in a refugee camp in Maban, South Sudan (2012–2013), where overall anti-HEV IgG prevalence was 24.1% [10]. The difference in the age-stratified prevalence estimates between Kitgum, Maban and Am Timan outbreaks supports the hypothesis that there were higher levels of immunity already present in Am Timan prior to the outbreak.

The underlying immunity for HEV infection in Am Timan before the outbreak is also evidenced in our study results which showed that the proportion of symptomatic infections was significantly lower in persons with past HEV infections compared to persons with recent infections. In addition, our calculations based on self-reported AJS estimate that 54.8% of Am Timan population was susceptible to HEV when the outbreak started. Both sets of information strengthen our hypothesis around existing high levels of HEV endemicity in Am Timan prior to this outbreak. Finally, seropositivity results for HEV in a study in Chad between 1993 and 1994 showed that 22% of hospitalized persons without acute or fulminant hepatitis had antibodies for HEV infection [25], which indicates that high levels of endemicity were reported elsewhere in the country as well.

The higher level of immunity prior the outbreak might explain the limited impact this outbreak had on the population in Am Timan in comparison to Maban or Kitgum (smaller magnitude, hospitalization rate and CFR) [20]. However, immunity levels alone cannot be the explanation. Following the Maban seroprevalence study where 54.3% of the population had been identified as still susceptible, the outbreak took hold in the refugee camps leading to a dramatic increase in cases of AJS [19]. The precise reason for this difference in outbreak dynamic is difficult to explain. A plausible reason might be that the combination of underlying population-based immunity for HEV and a reduced community-based transmission slowed the spread of the outbreak in Am Timan resulting in a less dramatic outbreak. The reduced transmission could have been facilitated by an impact of the control measures which included extensive bucket chlorination activities for most public water sources, hygiene kit distributions across all estimated 11,000 households during the outbreak and good coverage of hygiene promotion messages [26]. An alternative or complementary explanation for the reduced transmission could be the open/urban setting itself. A 2017 study by Azman, et al. showed that the risk of HEV infection in a camp was 2-fold higher than in people’s home states, even though the difference was not statistically significant [27].

An interesting finding concerned the identified seroprevalence for recent infections in women who reported that they were pregnant at the time of the outbreak. Even though we anticipate that the number of self reported pregnancies was likely underestimated, the seroprevalence of recent HEV infection in this group of women was lower than that in the general population. This finding suggests that the more severe clinical course of HEV infection, which has been so frequently observed in previous outbreaks [3, 4], is not due to the higher prevalence of infection in this population group, but is due to the pathophysiological effects of HEV infection during pregnancy.

The fact that 55.8% of recent HEV cases were clustered in a household suggests that transmission might have taken place at household level. The implication of transmission of HEV at household level has also been suggested in previous HEV outbreaks [5, 8, 28], despite these being in camp settings and not open urban settings such as Am Timan. Surveillance data from Am Timan outbreak support this hypothesis as having another person with jaundice in the household and having more than five people in the household were predictive of being a confirmed HEV case [18]. This current study also identified household level factors as being associated with recent HEV infections, such as sharing a latrine with several households and not systematically using soap for handwashing. Even though we were unable to identify clear HEV transmission routes in this study, these risk factors suggest that the virus could have spread when contaminated hands came into contact with stored water, or with a communal food plates or drinking vessels. In Am Timan, cross-contamination of water or food seems plausible as the majority of households uses wide-mouthed ceramic jars to store their water [26] (which facilitates water contamination) and communal eating vessels are commonly used.

We showed that persons living in households where animals could access the family sleeping area were more at risk of having a recent HEV infection. This could either be related to high risk behaviors in animal owners that expose them to environmental contamination with HEV or it could mean that animals are involved in the transmission of the virus. Although zoonotic transmission is well-recognized for genotype 3 and 4 of HEV, it has never been demonstrated for genotype 1 and 2 [1]. However, the possibility of animal involvement in HEV genotype 1 transmission should not be excluded and further explored.

The major limitation of this study is that it was a single serological survey conducted at the tail-end of an HEV outbreak. Therefore, we were unable to document the dynamics of anti-HEV seroprevalence in this affected population. The duration and magnitude of the IgM response in infected individuals has only been well documented for genotype 3 and 4 infections and even less is known about the immune response after asymptomatic infection. The IgM level following an acute infection of HEV genotype 4 was reported to remain high for up to 2 months in most patients and to drop below detection levels after 32 weeks (8 months) [29]. Whether the immune response for genotype 1e would be similar is unknown. However, it seems likely that we missed a proportion of individuals who were infected early during the current outbreak but for whom the IgM had waned by time this study was implemented (7 to 8 months after the first outbreak alert). This is also suggested by the relatively weak IgM reactivity in most of the IgM positive samples. While samples from clinically ill HEV patients collected early during the outbreak (not as part of the study) almost always showed a saturated signal (S/CO > 15), the majority (53.5%) of the IgM positive study samples only displayed reactivity between the cutoff and 2.5 times the cutoff of the assay (Additional file 4: Figure S1). We were therefore unable to attribute past HEV infections to the outbreak or to past exposures. Consequently, the impact of this outbreak could not be assessed reliably, the underlying endemicity could only be hypothesized and risk factors could have been missed. It is also possible that we underestimated the overall seroprevalence of anti_HEV antibodies in the event that persons with acute HEV infections sought care outside of Am Timan at the time of the survey. All exposures to potential HEV infection risk factors were self-reported in this survey and thus, subjected to social-desirability bias and recall bias. Since we assessed water, sanitation and hygiene practices at the time of the interview, they might have changed during the course of the outbreak and therefore, some risk factors might have been missed. Finally, HEV outbreaks have been previously documented in semi-nomadic populations [30] and we know that part of the population in Am Timan also has this characteristic. Unfortunately, the study design was not adapted to capture these population groups and thus, we were not able to assess HEV endemicity or the risk factors associated to infection in this specific sub-population.

## Conclusion

The high underlying immunity for HEV infection in the population of Am Timan before the start of the outbreak probably contributed, among other unidentified factors, to the limited impact of it (low AR, low hospitalization rate, low CFR). Therefore, we recommend implementing rapid serosurveys at the start of future outbreaks as it would facilitate risk assessment around the potential scale and impact of the outbreak. The development of better tools for simplified sample collection such as dried blood spots or oral swabs would be hugely beneficial for this. This study has again illustrated the role that household level factors play in the ongoing transmission of this virus. Based on our risk factor analysis, we recommend that control measures in future outbreaks continue to be focused on providing safe water and improving the level of personal hygiene at household level (i.e. hygiene kit/soap distributions and intensive household level hygiene promotion and education activities). In addition, we suggest considering the implementing of sanitation interventions to decrease the spread of HEV between households via latrine sharing.

## Additional files

Additional file 1: (French version). Questionnaire household and individual French. Household and individual questionnaires used during the HEV seroprevalence survey in Am Timan (Chad), 2017. (DOCX 56 kb)

Additional file 2: (English version). Questionnaire household and individual English. Household and individual questionnaires used during the HEV seroprevalence survey in Am Timan (Chad), 2017. (DOCX 57 kb)

Additional file 3: **Table S1.** Adjusted seroprevalence of HEV infection stratified by age group (*N* = 1494). This is the numeric data associated with Fig. 2 in the manuscript. It shows the adjusted seroprevalence estimates by age group for recent infection (anti-HEV IgM+ / IgG±), past infection with HEV (anti-HEV IgM- / IgG+) and susceptibility for HEV infection (anti-HEV IgM- / IgG-) in Am Timan during this seroprevalence survey. (DOCX 14 kb)

Additional file 4: **Figure S1.** Reactivity of IgM positive samples during the 2016–2017 outbreak in Am Timan, Chad. this figure contains reactivity data and the proportion of samples with optical density (OD) and cut-off (CO) ratios for anti-HEV IgM positive samples tested during the 2016–2017 HEV outbreak in Am Timan (Chad), which preceded the seroprevalence study discussed in this manuscript. (TIF 1831 kb)

## Abbreviations

95%CI: 95% Confidence Interval
AJS: Acute Jaundice Syndrome
AR: Attack Rate
CFR: Case Fatality Rate
EDTA: Ethylene Diamine Tetra acetic acid
EIA: Enzyme Immuo Assay
HEV: Hepatitis E Virus
IDPs: Internally Displaced Persons
IgG: Immunoglobulin G
IgM: Immunoglobulin M
MSF: Médecins Sans Frontières
ODK: Open Data Kit
PR: Prevalence Ratio
RDT: Rapid Diagnostic Test
