# Supplementary material for: Seroprevalence and risk factors of recent infection with hepatitis E virus during an acute outbreak in an urban setting in Chad, 2017
Source: BMC Infect Dis. 2018 Jun 26;18:287. doi: 10.1186/s12879-018-3194-6 (PMC6020170; doi:10.1186/s12879-018-3194-6)
Supplement: Supplementary file 1 — (French version). Questionnaire household and individual French. Household and individual questionnaires used during the HEV seroprevalence survey in Am Timan (Chad), 2017. (DOCX 56 kb) [file 12879_2018_3194_MOESM1_ESM.docx]

| **Additional file 1 : Household and individual questionnaire, French**  **Questionnaire sur les habitations – Enquête sérologique sur l’HEV** | | | | | |  |
| --- | --- | --- | --- | --- | --- | --- |
| **Numéro de la famille** |  | **Equipe** |  | **Date:** |  | |

| **CONSTITUTION DU LOGEMENT** | | | |
| --- | --- | --- | --- |
| **N°** | **Questions** | **Codage Catégories** | **Sauts** |
| **1** | Combien de personnes vivent dans votre foyer actuellement? | __________ |  |

| **DRINKING WATER** | | | | |
| --- | --- | --- | --- | --- |
| **N°** | **Questions** | **Codage Catégories** | **Sauts** | |
|  |  |  | |  |
| **2** | Quelle est la **principale** source d’approvisionnement en eau potable pour les membres de cette famille?  **CHOISIR UNE SEULE RESPONSE** | Eau du robinet  Robinet à l’intérieur de la maison 🞏  Robinet dans la cours, dans la concession, sur un terrain 🞏  Robinet du voisin 🞏  Pompe publique/colonne d’alimentation🞏  Puits tubé, forage 🞏  Puits creusé  Puits aménagé 🞏  Puits non-aménagé 🞏  Eau de source  Source aménagée 🞏  Source non-aménagée 🞏  Collecte d’eau de pluie 🞏  Cuve de camion 🞏  Charrette avec petit réservoir/ 🞏  Eau de surface (fleuve, rivière, barrage, lac, étang, canalisation, voie d’irrigation) 🞏  Eau embouteillée 🞏  Autre (Spécifier) ______________________ 🞏 |  | |
| **3** | Traitez-vous votre eau? | Oui 1  Non 2 | **🡪Q5** | |
| **4** | Comment traitez-vous votre eau avant de la boire?  **VERIFIER TOUTES LES REPONSES ENTREES** | Bouillir 🞏  Ajouter de la javel / du chlore 🞏  Filtrer à travers un linge 🞏  Utiliser un filtre à eau (céramique, sable) 🞏  Laisser se décanter 🞏  Autre (Spécifier) ____________________ 🞏  Aucune idée 🞏 |  | |

| **EAU POTABLE** | | | |
| --- | --- | --- | --- |
| **N°** | **Questions** | **Codage Catégories** | **Sauts** |
| **5** | Buvez-vous parfois de l’eau de surface (stagnante, drain, étang, fleuve, rivière) ? | Oui 1  Non 2 | **🡪Q6**  **🡪Q8** |
| **6** | Avant de boire de l’eau de surface, la traitez-vous? | Oui 1  Non 2 | **🡪Q7**  **🡪Q8** |
| **7** | Si oui, comment traitez-vous votre eau de surface avant de la boire?  **VERIFIER TOUTES LES REPONSES ENTREES** | Bouillir 🞏  Ajouter de la javel /du chlore 🞏  Filtrer à travers un linge 🞏  Utiliser un filtre à eau (céramique, sable) 🞏  Laisser se décanter 🞏  Autre (Spécifier) ____________________🞏  Aucune idée 🞏 |  |

| **STOCKAGE DE L’EAU** | | | |
| --- | --- | --- | --- |
| **N°** | **Questions** | **Codage Catégories** | **Sauts** |
| **8** | Avez-vous des récipients dans lesquels stocker de l’eau? | Oui 1  Non 2 | **🡪Q9**  **🡪Q13** |
| **9** | Combien de récipients de stockage avez-vous? | Entrer le nombre de récipients que vous pouvez voir:  __________ |  |
| **10** | Puis-je voir le(s) récipient(s) de stockage d’eau? | Oui 1  Non 2 | **🡪Q11**  **🡪Q13** |
| **11** | Quelle est votre capacité de stockage totale (en litres) d’eau? | Entrer le nombre de récipients que vous pouvez voir:  __________ |  |
| **12** | **OBSERVER ET NOTER les caractéristiques suivantes du réservoir d’eau de l’habitation** | \| **Caractéristiques** \| **Nombre** \| \| --- \| --- \| \| Translucide \|  \| \| Ouverture étroite (<10cm) \|  \| \| Propre (aucune saleté visible à l’intérieur) \|  \| \| Couvercle/Couvre de manière sûre \|  \| \| Possède un robinet \|  \| \| Verser pour boire \|  \| \| Puiser dans le récipient d’eau pour boire \|  \| |  |

| **UTILISATION DES TOILETTES** | | | |
| --- | --- | --- | --- |
| **N°** | **Questions** | **Codage catégories** | **Sauts** |
| **13** | Quel est le principal type de toilettes utilisées par les membres de votre famille**?**  **VERIFIER TOUTES LES REPONSES ENTREES** | Chasse d’eau / verser de l’eau  Système d’évacuation vers les égouts 🞏  Evacuation vers une fosse septique 🞏  Evacuation vers une fosse (latrine) 🞏  Evacuation ailleurs 🞏  Evacuation vers un endroit inconnu/pas sûr/Ignore où 🞏  Latrine  Latrine ventilée améliorée (LVA)  🞏  Latrine avec dalle 🞏  Latrine sans dalle / ouverte 🞏  Toilettes de compost 🞏  Seau 🞏  Toilettes suspendues, latrine suspendue 🞏  Aucune installation, brousse ou champs 🞏  Autre (*Spécifier*)­­­­­­­­­­­­­­­­­­­­­­_____________________🞏 |  |
| **14** | Partagez-vous les mêmes toilettes avec d’autres foyers? | Oui 1  Non 2 |  |
| **15** | Puis-je voir ces toilettes ? | Oui 1  Non 2 |  |
| **16** | Ces toilettes ont-elles: | ***Vérifier la présence de:***  Couvercle 🞏  Matières fécales visibles/saleté 🞏  Lave-mains SANS savon 🞏  Lave-mains AVEC savon 🞏 |  |
| **17** | Où vos enfants font-ils leurs besoins /où vont-ils aux toilettes? | Latrine 🞏  Brousse/ en plein air 🞏  Etang / fleuve 🞏  Autres (*spécifier*) 🞏 |  |
| **18** | Comment gérez-vous et comment vous débarrassez-vous des matières fécales de vos enfants? | Vous les laissez à l’air libre 🞏  Vous les couvrez avec des feuilles 🞏  Vous les couvrez avec de la terre 🞏  Vous les jetez dans les latrines 🞏 |  |

| **LAVAGE DES MAINS** | | | |
| --- | --- | --- | --- |
| **N°** | **Questions** | **Codage Catégories** | **Sauts** |
| **19** | Avez-vous du savon ? | Oui 1  Non 2 |  |
| **20** | Puis-je voir votre savon? | Oui 1  Non 2 |  |
| **21** | **OBSERVER ET ENREGISTRER LA QUANTITE DE SAVON VUE** | Entrer le nombre total de savons que vous pouvez voir:  __________ |  |

| LAVAGE DES MAINS | | | |
| --- | --- | --- | --- |
| **N°** | **Questions** | **Codage Catégories** | **Sauts** |
| **22** | Quand lavez-vous vos mains?  (choisir plus d’une réponse)  **VERIFIER TOUTES LES REPONSES APPLICABLES** | Avant de manger 🞏  Avant de cuisiner/de préparer les repas 🞏  Avant de nourrir un enfant 🞏  Avant de prier 🞏  Après être allé(e) aux toilettes 🞏  Lorsqu’elles sont sales 🞏  Après avoir nettoyé les matières fécales d’un enfant 🞏  Après avoir travaillé dehors 🞏  Lorsque vous prenez votre bain 🞏  Vous n’êtes pas sûr(e)/vous ne savez pas 🞏  Autres (spécifier)___________________ 🞏 |  |
| **23** | Comment lavez-vous vos mains (choisir plus d’une option) | Avec de l’eau et de la cendre 🞏  Avec de l’eau et du savon 🞏  Dans une cuvette de lavage commune 🞏  Avec seulement de l’eau 🞏  Autres (*spécifier*)___________________🞏 |  |
| **24** | **Observer comment/ où ils lavent leurs mains et décrivez ici** |  |  |
| **25** | Comment l’avez-vous les mains de vos enfants (choisir plus d’une option) | Avec de l’eau et de la cendre ………………. 🞏  Avec de l’eau et du savon 🞏  Dans une cuvette de lavage commune 🞏  Avec seulement de l’eau 🞏  Autres (*spécifier*) __________________🞏 |  |

| **CAS DE JAUNISSE SURVENUS DANS LA FAMILLE** | | | |
| --- | --- | --- | --- |
| **N°** | **Questions** | **Codage Catégories** | **Sauts** |
| **26** | Une personne de votre foyer a-t-elle présenté des symptômes de jaunisse/yeux jaune depuis le mois de Septembre? | Oui 1  Non 2 | **🡪Q28** |
| **27** | Si oui, combien de personnes?  *Veillez à recueillir ces informations dans le questionnaire individuel pour le prélèvement des échantillons* | Entrez le nombre de personnes ici:  __________ |  |
| **28** | Depuis Septembre, des personnes de votre foyer sont-elles décédées de la maladie des yeux jaune? | Oui 1  Non 2 | **🡪Q28** |
| **29** | Si oui, combien de personnes?  *Veillez à recueillir ces informations dans le questionnaire individuel pour le prélèvement des échantillons* | Pour chaque personne, recueillez l’âge et le sexe ainsi que la date approximative du décès:  ______________________________________  ______________________________________  ______________________________________ |  |

| **POSSESSION D’ANIMAUX DOMESTIQUES** | | | |
| --- | --- | --- | --- |
| **N°** | **Questions** | **Codage Catégories** | **Sauts** |
| **30** | Possédez-vous des poulets, des chiens, des chats, des vaches, des chèvres ou tout autre animal domestique qui vivent dans votre foyer ? | Oui 1  Non 2 |  |
| **31** | Si oui, quel animal domestique possédez-vous?  (vérifier la présence) | Poulets 🞏  Chiens 🞏  Chats 🞏  Vaches 🞏  Chèvres 🞏  Autres (*spécifier*) 🞏 |  |
| **32** | Quels animaux dorment dans votre foyer? | Poulets 🞏  Chiens 🞏  Chats 🞏  Vaches 🞏  Chèvres 🞏  Autres (*spécifier*) 🞏 |  |
| **33** | Quels animaux dorment à l’extérieur de votre foyer? | Poulets 🞏  Chiens 🞏  Chats 🞏  Vaches 🞏  Chèvres 🞏  Autres (*spécifier*) 🞏 |  |

**Questionnaire individuel – séroprévalence Hépatite E, Am Timam, Tchad**

| **N° chef de ménage :** |  | **Équipe** |  | **Date :** |  |
| --- | --- | --- | --- | --- | --- |

| **N° chef de ménage** | **ID** | **Sexe** | **Âge** | **Si c’est une femme, est-elle enceinte ?** | **Consentement éclairé** | **Consentement éclairé pour enfants**  **8 - 18 ans** | **Depuis septembre, avez-vous constaté un de ces trois symptômes simultanément ?** | **Si oui, à quelle date ?** | **Si oui, avez-vous cherché à obtenir de l’aide à l’hôpital ? Si oui, où ?** | **Si oui, étiez-vous enceinte à ce moment-là ?** | **Prélèvement sanguin** | **Prélèvement DBS** | **Prélèvement oral** | **Test RDT malaria** | **Si test RDT positif, traitement administré ?** |
| --- | --- | --- | --- | --- | --- | --- | --- | --- | --- | --- | --- | --- | --- | --- | --- |
| *Complétez* |  | *0 = homme*  *1 = femme* | *Mois (M)*  *Années (A)* | *1= oui*  *2= non*  *3 = ne sait pas* |  |  | *Jaunisse, fièvre, malaise* | *Date d’admission jj/mm/aa* | *1=Hôpital*  *2=Centre de santé*  *3=Traditionnel*  *4= Autre (préciser)* | *1= oui*  *2= non*  *3 = ne sait pas* |  |  |  | *1= pos.*  *2= nég.* | *1= oui, au niveau du chef de ménage*  *2= oui, redirigé(e) vers l’hôpital*  *3= non, ne voulait pas*  *4= Autre (préciser)* |
|  | **001** |  |  |  | **🞏** | **🞏** |  |  |  |  | **🞏** | **🞏** | **🞏** |  |  |
|  | **002** |  |  |  | **🞏** | **🞏** |  |  |  |  | **🞏** | **🞏** | **🞏** |  |  |
|  | **003** |  |  |  | **🞏** | **🞏** |  |  |  |  | **🞏** | **🞏** | **🞏** |  |  |
|  | **004** |  |  |  | **🞏** | **🞏** |  |  |  |  | **🞏** | **🞏** | **🞏** |  |  |
|  | **005** |  |  |  | **🞏** | **🞏** |  |  |  |  | **🞏** | **🞏** | **🞏** |  |  |
|  | **006** |  |  |  | **🞏** | **🞏** |  |  |  |  | **🞏** | **🞏** | **🞏** |  |  |
|  | **007** |  |  |  | **🞏** | **🞏** |  |  |  |  | **🞏** | **🞏** | **🞏** |  |  |
|  | **008** |  |  |  | **🞏** | **🞏** |  |  |  |  | **🞏** | **🞏** | **🞏** |  |  |
|  | **009** |  |  |  | **🞏** | **🞏** |  |  |  |  | **🞏** | **🞏** | **🞏** |  |  |
|  | **010** |  |  |  | **🞏** | **🞏** |  |  |  |  | **🞏** | **🞏** | **🞏** |  |  |

| **N° chef de ménage :** |  | **Équipe** |  | **Remettez un exemplaire de ce formulaire au chef de ménage**  **(Copie du texte ci-dessus)** |
| --- | --- | --- | --- | --- |

| **Numéro chef de ménage** | **ID** | **Sexe** | **Âge** | **Nom de la personne** |
| --- | --- | --- | --- | --- |
| *Complétez* |  | *0 = homme*  *1 = femme* | *0 = 0-11 mois*  *1 = 1 an ; 2 = 2 ans etc.* | *À ajouter UNIQUEMENT sur demande du chef de ménage* |
|  | **001** |  |  |  |
|  | **002** |  |  |  |
|  | **003** |  |  |  |
|  | **004** |  |  |  |
|  | **005** |  |  |  |
|  | **006** |  |  |  |
|  | **007** |  |  |  |
|  | **008** |  |  |  |
|  | **009** |  |  |  |
|  | **010** |  |  |  |
