# Supplementary material for: Seroprevalence and risk factors of recent infection with hepatitis E virus during an acute outbreak in an urban setting in Chad, 2017
Source: BMC Infect Dis. 2018 Jun 26;18:287. doi: 10.1186/s12879-018-3194-6 (PMC6020170; doi:10.1186/s12879-018-3194-6)
Supplement: Supplementary file 2 — (English version). Questionnaire household and individual English. Household and individual questionnaires used during the HEV seroprevalence survey in Am Timan (Chad), 2017. (DOCX 57 kb) [file 12879_2018_3194_MOESM2_ESM.docx]

| **Additional file 2: Household and individual questionnaire English**  **Household Questionnaire – Serosurvey HEV** | | | | | |  |
| --- | --- | --- | --- | --- | --- | --- |
| **HH Number** |  | **Team** |  | **Date:** |  | |

| **HOUSEHOLD MAKE UP** | | | |
| --- | --- | --- | --- |
| **N°** | **Questions** | **Coding Categories** | **Skips** |
| **1** | How many people live in your household at the moment? | __________ |  |
| **2** | How many people in your household were absent from your household for a period of 2 weeks in the last six months? | __________ |  |
| **3** | How many persons eligible to participate in the study are actually present at the time of the interview  **OBSERVE** the persons eligible in the household and those that were not absent for two weeks in the last six months | __________ |  |

| **DRINKING WATER** | | | | |
| --- | --- | --- | --- | --- |
| **N°** | **Questions** | **Coding Categories** | **Skips** | |
|  |  |  | |  |
| **4** | What is the **main** source of drinking water for members of this household?  **CHECK ONLY ONE RESPONSE** | Piped water  Piped into dwelling 🞏  Piped into compound, yard or plot 🞏  Piped to neighbour 🞏  Borehole with pump 🞏  Borehole with open water/water salesmen 🞏  Tap stand 🞏  Traditional well without set up 🞏  Surface water (stagnant, river, stream, dam, lake, pond, canal, irrigation channel, hole in the Wadi) 🞏  Bottled water 🞏  Doesn’t know 🞏  Other (Specify)______________________ 🞏 |  | |
| **5** | Is the main water source you use currently chlorinated by MSF teams? | Yes 1  No 2  Doesn’t know 88 |  | |
| **6** | Do you treat your water in your home/household? | Yes 1  No 2 | **🡪Q7**  **🡪Q8** | |
| **7** | How do you treat the water before drinking?  **DO NOT READ ALL THE OPTIONS**  **CHECK ALL RESPONSES MENTIONED** | Boil 🞏  Add bleach/chlorine 🞏  Strain it through a cloth 🞏  Use water filter (Ceramic, sand) 🞏  Let it stand and settle 🞏  Other (Specify)_____________________ 🞏  Don’t know 🞏 |  | |
|  | | | | |
|  |  |  |  | |
| **8** | Do you sometimes drink surface water (standing, drainage, pond, river, stream) ? | Yes 1  No 2 | **🡪Q6**  **🡪Q8** | |
| **9** | If you drink surface water, do you treat it first? | Yes 1  No 2 | **🡪Q7**  **🡪Q8** | |
| **10** | If yes, how do you treat the surface water before drinking?  **CHECK ALL RESPONSES MENTIONED** | Boil 🞏  Add bleach / chlorine 🞏  Strain it through a cloth 🞏  Use water filter (ceramic, sand) 🞏  Let it stand and settle 🞏  Other (Specify)______________________ 🞏  Don’t know 🞏 |  | |

| **WATER STORAGE** | | | |
| --- | --- | --- | --- |
| **N°** | **Questions** | **Coding Categories** | **Skips** |
| **11** | Do you have any water storage containers | Yes 1  No 2 | **🡪Q12**  **🡪Q16** |
| **12** | Can I see your containers? | Yes 1  No 2 | **🡪Q13**  **🡪Q16** |
| **13** | Number of water containers | __________ |  |
| **14** | **OBSERVE AND RECORD the following characteristics of the water storage in the household** | \| **Characteristic** \| **Number** \| \| --- \| --- \| \| Translucent \|  \| \| Narrow mouth (<10cm) \|  \| \| Clean (no visible dirt inside) \|  \| \| Lid/secure fitting cover \|  \| \| Has a tap \|  \| \| Water is taken by pouring \|  \| \| Dipping into container for water \|  \| |  |
| **15** | **Estimate the total water storage capacity of the combined number of water storage containers.**  **OBSERVE** | _____________ |  |

| **TOILET USE** | | | |
| --- | --- | --- | --- |
| **N°** | **Questions** | **Coding Categories** | **Skips** |
| **16** | What is the **main** type of toilet facility used by members of your household**?**  **CHECK ONE SINGLE RESPONSE** | Flush toilet 🞏    Pit latrine with slab 🞏  Pit latrine without slab/open pit 🞏  No facility, bush, field 🞏  Other (Specify)­­­­­­­­­­­­­­­­­­­­­­_____________________🞏 |  |
| **17** | Is the toilet shared with other households? | Yes 1  No 2 |  |
| **18** | May I see the toilet | Yes 1  No 2 |  |
| **19** | Does the toilet have: | ***Check if present:***  Cover 🞏  Visible faeces/dirty 🞏  Handwashing point NO soap 🞏  Handwashing point WITH soap 🞏 |  |
| **20** | Where do your children defecate/go to the toilet?  **DO NOT READ THE OPTIONS. MARK ALL RESPONSES NECESSARY.**  This refers to children that cannot go to the toilet themselves. | Latrine 🞏  Bush/open defecation 🞏  Pond/river 🞏  Other (specify) 🞏 |  |
| **21** | How do you handle/dispose of your children’s faeces and dispose of them? | Leave them uncovered 🞏  Cover up with leaves 🞏  Cover up with soil 🞏  Put them in the latrine 🞏 |  |

| **HANDWASHING** | | | |
| --- | --- | --- | --- |
| **N°** | **Questions** | **Coding Categories** | **Skips** |
| **22** | Do you have soap ? | Yes 1  No 2 | **-->Q23**  **🡪 Q25** |
| **23** | May I see your soap | Yes 1  No 2 | **-->Q24**  **🡪 Q25** |
| **24** | **OBSERVE AND RECORD THE NUMBER OF SOAP SEEN** | Enter the total number of soaps you can see:  __________ |  |

| **HANDWASHING** | | | |
| --- | --- | --- | --- |
| **N°** | **Questions** | **Coding Categories** | **Skips** |
| **25** | When do you wash your hands?  (select more than one option)  **Do not read the options**  **CHECK ALL THAT APPLY** | Before eating 🞏  Before cooking/preparing food 🞏  Before feeding a child 🞏  Before prayer 🞏  After going to the toilet 🞏  When dirty 🞏  After handling a child’s stool/faeces 🞏  After working outside 🞏  When bathing 🞏  Don’t know 🞏  Other (specify) _____________________🞏 |  |
| **26** | How do you wash your hands ?  **Do not read the options. Mark all responses required** | Water with ash 🞏  Water with soap 🞏  Communal handwashing basin 🞏  Water only 🞏  Other (specify) _____________________🞏 |  |
| **27** | **Describe how they wash their hands** | Correctly 🞏  Incorrect 🞏 |  |
| **28** | How do you wash your children’s hands  **Do not read the options**  **CHECK ALL THAT APPLY** | Water with ash 🞏  Water with soap 🞏  Communal handwashing basin 🞏  Water only 🞏  Other (specify) _____________________🞏 |  |

| **HISTORY OF JAUNDICE IN THE HOUSEHOLD** | | | |
| --- | --- | --- | --- |
| **N°** | **Questions** | **Coding Categories** | **Skips** |
| **29** | Since Tabaski (September) has anyone in your household displayed symptoms of jaundice/yellow eyes ? | Yes 1  No 2 | **🡪Q30🡪Q33** |
| **30** | How many people?  *Make sure you capture this information in the individual questionnaires for the sample taking* | __________ |  |
| **31** | Since Tabaski (September) has anyone in your household died of yellow eyes at home? | Yes 1  No 2 | **🡪Q32**  **🡪Q33** |
| **32** | Hhow many people? | For each person capture age and sex and if the person was pregnant and approximate date of death:  ______________________________________  ______________________________________  ______________________________________ |  |

| **DOMESTIC ANIMAL OWNERSHIP** | | | |
| --- | --- | --- | --- |
| **N°** | **Questions** | **Coding Categories** | **Skips** |
| **33** | Do you own any chickens, dogs, cats, donkeys, cows, goats or other animals that live in the household? | Yes 1  No 2 | **🡪Q31**  **🡪Q37** |
| **34** | If yes, which animals do you have?  (check the applicable and try and verify their presence) | Chickens 🞏  Dogs 🞏  Cats 🞏  Cows 🞏  Goats 🞏  Donkeys 🞏  Others (specify) 🞏 |  |
| **35** | Which animals sleep inside your household | Chickens 🞏  Dogs 🞏  Cats 🞏  Cows 🞏  Goats 🞏  Donkeys 🞏  Others (specify) 🞏 |  |
| **36** | Which animals sleep outside your household | Chickens 🞏  Dogs 🞏  Cats 🞏  Cows 🞏  Goats 🞏  Donkeys 🞏  Others (specify) 🞏 |  |
| **37** | Do you own camels? | Yes 1  No 2 |  |

**Individual Questionnaire – seroprevalence Hepatitis E, Am Timam, Chad**

| **HH NUM:** |  | **Team** |  | **Date:** |  |
| --- | --- | --- | --- | --- | --- |

| **ID** | **Does this person match the inclusion criteria (including having provided informed consent/assent)** | **Sexe** | **Age** | **If female, is she pregnant now ?** | **Since Tabaski/September, did you experience jaundice?** | **If yes, which date?** | **Since Tabaski/September, did you experience fever?** | **If yes, which date?** | **Since Tabaski/September, did you experience discomfort(malaise)?** | **If yes, which date?** | **If yes, did you seek care at the hospital? If yes, where?** | **If not, why not** | **If yes, were you pregnant at the time?** | **Whole blood taken** | **DBS Taken** | **Oral swab taken** | **Malaria RDT** | **If RDT positive, treatment given?** |
| --- | --- | --- | --- | --- | --- | --- | --- | --- | --- | --- | --- | --- | --- | --- | --- | --- | --- | --- |
|  | *1= yes*  *2= no* | *0 = Male*  *1 = Female* | *Months (M)*  *Years (Y)* | *1= yes*  *2= no*  *3 = not known* | *1= yes*  *2= no*  *3 = not known* | *Enter date dd/mm/yy* | *1= yes*  *2= no*  *3 = not known* | *Enter date dd/mm/yy* | *1= yes*  *2= no*  *3 = not known* | *Enter date dd/mm/yy* | *1=Hospital*  *2=Health centre*  *3=Traditional*  *4=Pharmacy*  *5=Boutique*  *77= Other (specify)* | *1= no money ; 2=no time ; 3=too far ; 4=not sick enough ; 5=no trust in health care staff ; 6=required to wait too long ; 7=embarassed ; 77= other* | *1= yes*  *2= no*  *3 = not known* |  |  |  | *1= pos*  *2= neg* | *1= yes, at HH level*  *2= yes, referred to hospital*  *3=No, didn’t want it*  *4=Other (specify)* |
| **001** |  |  |  |  |  |  |  |  |  |  |  |  |  | **🞏** | **🞏** | **🞏** |  |  |
| **002** |  |  |  |  |  |  |  |  |  |  |  |  |  | **🞏** | **🞏** | **🞏** |  |  |
| **003** |  |  |  |  |  |  |  |  |  |  |  |  |  | **🞏** | **🞏** | **🞏** |  |  |
| **004** |  |  |  |  |  |  |  |  |  |  |  |  |  | **🞏** | **🞏** | **🞏** |  |  |
| **005** |  |  |  |  |  |  |  |  |  |  |  |  |  | **🞏** | **🞏** | **🞏** |  |  |
| **006** |  |  |  |  |  |  |  |  |  |  |  |  |  | **🞏** | **🞏** | **🞏** |  |  |
| **007** |  |  |  |  |  |  |  |  |  |  |  |  |  | **🞏** | **🞏** | **🞏** |  |  |
| **008** |  |  |  |  |  |  |  |  |  |  |  |  |  | **🞏** | **🞏** | **🞏** |  |  |
| **009** |  |  |  |  |  |  |  |  |  |  |  |  |  | **🞏** | **🞏** | **🞏** |  |  |
| **010** |  |  |  |  |  |  |  |  |  |  |  |  |  | **🞏** | **🞏** | **🞏** |  |  |

| **HH NUM:** |  | **Team** |  | **Give a copy of this form to the Head of the Household**  **(*Copy from Above*)** |
| --- | --- | --- | --- | --- |

| **HH Number** | **ID** | **Sexe** | **Age** | **Name of the person** |
| --- | --- | --- | --- | --- |
| *Fill in* |  | *0 = Male*  *1 = Female* | *Months (M) ; Years (Y)* | *To be added ONLY on request of the Head of Household* |
|  | **001** |  |  |  |
|  | **002** |  |  |  |
|  | **003** |  |  |  |
|  | **004** |  |  |  |
|  | **005** |  |  |  |
|  | **006** |  |  |  |
|  | **007** |  |  |  |
|  | **008** |  |  |  |
|  | **009** |  |  |  |
|  | **010** |  |  |  |
