# Supplementary material for: Seroprevalence and risk factors of recent infection with hepatitis E virus during an acute outbreak in an urban setting in Chad, 2017
Source: BMC Infect Dis. 2018 Jun 26;18:287. doi: 10.1186/s12879-018-3194-6 (PMC6020170; doi:10.1186/s12879-018-3194-6)
Supplement: Supplementary file 3 — Table S1. Adjusted seroprevalence of HEV infection stratified by age group (N = 1494). This is the numeric data associated with Fig. 2 in the manuscript. It shows the adjusted seroprevalence estimates by age group for recent infection (anti-HEV IgM+ / IgG±), past infection with HEV (anti-HEV IgM- / IgG+) and susceptibility for HEV infection (anti-HEV IgM- / IgG-) in Am Timan during this seroprevalence survey. (DOCX 14 kb) [file 12879_2018_3194_MOESM3_ESM.docx]

**Additional file 3: Table S1: Adjusted seroprevalence of HEV infection stratified by age group (N=1,494)**

| Age | Total | Recent HEV infection^a^ | | Past HEV infection^b^ | | HEV susceptible^c^ | |
| --- | --- | --- | --- | --- | --- | --- | --- |
| Years | N | n | % (95%CI) | n | % (95%CI) | n | % (95%CI) |
| <5 | 258 | 33 | 12.6% (8.7-17.9) | 60 | 23.5% (18.3-29.7) | 165 | 63.9% (57.4-70.0) |
| 5-14 | 531 | 42 | 7.9% (5.6-11.0) | 364 | 68.6% (62.7-73.9) | 125 | 23.5% (18.6-29.3) |
| ≥15 | 705 | 29 | 4.3% (2.9-6.3) | 537 | 75.9% (72.1-79.2) | 139 | 19.9% (16.8-23.3) |
| Total | 1,494 | 104 | 7.7% (6.2-9.6) | 961 | 59.6% (56.3-62.8) | 429 | 32.7% (29.6-35.9) |

^a^ anti-HEV IgM+ / IgG±; ^b^ anti-HEV IgM- / IgG+; ^c^ anti-HEV IgM- / IgG-
