# Supplementary figures and images for: Seroprevalence and risk factors of recent infection with hepatitis E virus during an acute outbreak in an urban setting in Chad, 2017
Source: BMC Infect Dis. 2018 Jun 26;18:287. doi: 10.1186/s12879-018-3194-6 (PMC6020170; doi:10.1186/s12879-018-3194-6)

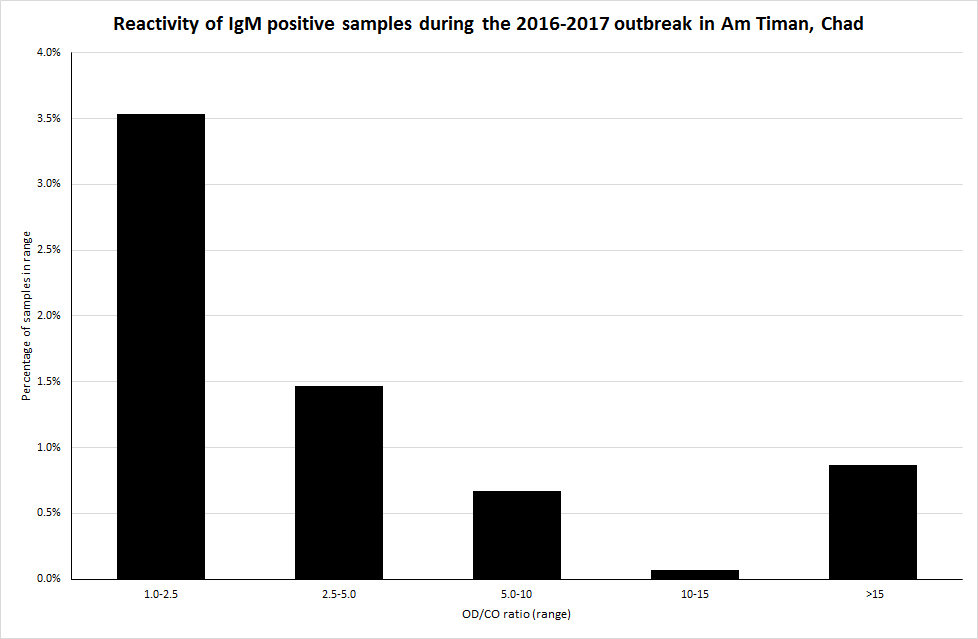

Supplement: Supplementary file 4 — Figure S1. Reactivity of IgM positive samples during the 2016–2017 outbreak in Am Timan, Chad. this figure contains reactivity data and the proportion of samples with optical density (OD) and cut-off (CO) ratios for anti-HEV IgM positive samples tested during the 2016–2017 HEV outbreak in Am Timan (Chad), which preceded the seroprevalence study discussed in this manuscript. (TIF 1831 kb) [file 12879_2018_3194_MOESM4_ESM.tif]
